# Autonomic cerebral vascular response to sildenafil in diabetic patient

**DOI:** 10.1186/1758-5996-4-2

**Published:** 2012-01-27

**Authors:** Fadhil G Al-Amran, Akeel AMH Zwain, Najah R Hadi, Ahmed M Al-Mudhaffer

**Affiliations:** 1 FRCS, FACS, Department of Surgery, postal code 474, Medical college Kufa University, Kufa Najaf street, Najaf, Iraq; 2 Department of Cardiovascular Physiology, postal code 474, Medical college Kufa University, Kufa Najaf street, Najaf, Iraq; 3 Department of Pharmacology, postal code 474, Medical college Kufa University, Kufa Najaf street, Najaf, Iraq; 4 Department of Pharmacology, postal code 474, Medical college Kufa University, Kufa Najaf street, Najaf, Iraq

**Keywords:** cerebrovascular reactivity, stroke, breath holding-hyperventilation test transcranial Doppler, sildenafil

## Abstract

**Background:**

Erectile dysfunction is a common problem in type 2 diabetic patients who are at higher risk of cerebrovascular events, and it's recorded with sildenafil, a drug which is primarily used for erectile dysfunction.

**Objectives:**

We tested the hypothesis whether or not sildenafil modulates cerebrovascular reactivity (CVR) in patients with type 2 diabetes mellitus.

**Methods:**

A total of 35 male participants were enrolled; eighteen with type 2 diabetes mellitus matched with seventeen normal individuals. Transcranial Doppler Ultrasonographic examination (TCD) was performed for all participants to insonate the middle cerebral artery (MCA) through a trans-temporal window. CVR was assessed by using breath holding (BH)-hyperventilation (HV) test, before and after oral 50 mg sildenafil; recordings were analyzed by using SPSS program version 12.

**Results:**

In normal individuals, sildenafil did not result in statistically significant change in breath holding index (BHI) from 0.91 ± 0.11 to 0.81 ± 0.09 and full range of vasodilatation (FVD) from (59.4% ± 6.3%) to (53.7% ± 4.9%). In diabetic patients, giving sildenafil resulted in significant increase in BHI (from 0.74 ± 0.14 to 1.03 ± 0.14) and FVD (from 60.2% ± 4.96% to 74% ± 4.8%), (p < 0.05).

**Conclusion:**

Sildenafil significantly improves CVR in type 2 diabetic patients but not in normal subjects.

## Introduction

Cerebrovascular reactivity (CVR) may be defined as the capability of intracerebral arterioles to constrict and to dilate ensuring constant cerebral blood flow despite changes in systemic blood pressure [1]. There are two types of cerebrovascular reactivity; first, mechanical cerebro-vascular reactivity which does not depend on nitric oxide (NO), second, chemical cerebrovascular reactivity which is endothelial in origin and NO dependent [2]. Carbon dioxide is a potent cerebral vasodilator, therefore, hypercapnea induces cerebral vasodilatation and hypercapnea or hyperoxia induces cerebral vasoconstriction [3,4]. CO_2_-NO axis is a cardinal pathway in the chemoregulation of cerebral blood flow (CBF) in humans; additionally, the results of Lavi et al, supported the hypothesis that endothelial dysfunction would result in impaired CO2 vasoreactivity (chemoregulation) of cerebral vasculature. This chemical cerebrovascular reactivity was described by Markus and Harrison who used breath holding/hyperventilation test for assessment of cerebrovascular reactivity. They measured the breath holding index (BHI) and full range of vasodilatation (FVD) of middle cerebral artery using transcranial Doppler; it has been shown that Breath holding is effective in inducing cerebral vasodilatation while hyperventilation is effective in inducing cerebral vasoconstriction.

It has also been demonstrated that endothelial function is more altered in type 2 diabetic patients than in type 1 diabetic patients, regardless of the differences in their blood glucose control levels [5], suggesting a possibility of involvements of other factors; a longer period of undetected blood glucose abnormalities, lipid alterations or decreased insulin sensitivity could be involved. Therefore, the initial acethylcholine-endothelium dependent dysfunction can be improved by the normalization of blood glucose control in type 1 diabetes mellitus, whereas it has never been observed in type 2 diabetes mellitus, Further, it has been reported that diabetes alters endothelial function and permeability of the blood-brain barrier, thus affecting micro-circulation and regional metabolism [5] with the result of impaired CVR (less increase in CBF).

In relevance to the current study, it is essential to shed some light on literature reports focusing on sildenafil and Cerebral Vasoreactivity: Sildenafil is known to have a potent and selective inhibitor of cGMP-specific PDE-5, the predominant isozyme that metabolizes cGMP in the corpus cavernosum of the penis [6]. cGMP is the second messenger of NO and, a principal mediator of smooth muscle relaxation and vasodilatation in the penis. By inhibiting the hydrolytic breakdown of cGMP, sildenafil prolongs the action of cGMP; this results in augmented smooth muscle relaxation, and hence, prolongation of the erection. Prior production of cGMP by NO released primarily from the nonadrenergic, noncholinergic (nitroxidergic) cavernosal nerves in response to sexual stimulation is required for sildenafil to be effective [7]. Both erectile dysfunction and cardiovascular disease share common risk factors, therefore, any drug used to treat erectile dysfunction may exert an impact upon patients with high incidence of diagnosed or covert cardiovascular disease. For this reason, it seems plausible to understand the effects of phosphodiesterase type 5 inhibitors and any other drugs used for the treatment of erectile dysfunction on the cardiovascular system [8]. In a study conducted on eleven patients with pulmonary hypertension, it is shown that sildenafil has beneficial effect on cerebrovascular reactivity indicated by improvement in neurovascular coupling in these patients [9]. In vitro, at high concentration, sildenafil was found to dilate isolated cerebral arteries of guinea pig; the response of vasodilatation was augmented by sodium nitroprusside pre-treatment and attenuated by endothelial removal or pharmacological inhibition of endogenous cGMP production [10,11]. It was proposed that such results support the lack of vascular response in human in vivo studies and may suggest a possible neural rather than vascular effect of sildenafil in migraine induction[11]. Aim of this study was to determine whether sildenafil modulates cerebrovascular reactivity (CVR) in patients with type 2 diabetes mellitus.

## Subjects and Methods

A total of thirty five subjects were participated in this study after obtaining their signed consent and formal approval of the Ethical Committee of the Faculty of Medicine of Kufa University. They were divided into two groups: Seventeen healthy volunteers, of a mean age of 45 ± 2.17 years (range: 33-58) and eighteen patients with type 2 DM, of a mean age of 50 ± 1.35 years (range: 42-57); the mean duration of diabetes from the time of diagnosis was 11.3 ± 6.35 years (range: 3-24). The study started from 26/10/2008 and ended in 3/7/2009. Patients were considered as diabetic according to World Health Organization definition of diabetes as fasting plasma glucose ≥ 7.0 mmol/L (126 mg/dL) or 2-hour post-load plasma glucose ≥ 11.1 mmol/L (200 mg/dL), according to WHO consultation, definition, diagnosis and classification of diabetes mellitus (1999). The selection of diabetic patients and normal subjects was according to certain inclusion and exclusion criteria (table 1, 2). For all patients meeting inclusion criteria, medical and surgical histories and demographic information were recorded. All participants were subjected for laboratory investigations including urine for albumin, fasting blood sugar, serum lipid profile, blood urea, creatinine, uric acid and liver function test. Full history and neurological motor, sensory and autonomic examination were undertaken to exclude diabetic neuropathy. For all participants, a full physical examination including heart rate, blood pressure measurements and a 12-lead electrocardiogram, were performed.

**Table 1:** Inclusion criteria

Criteria type	Inclusion criteria
Patient criteria	1. Male 2. Type 2 diabetes mellitus for diabetic group 3. Co-operative

TCD examination criteria	4. Sufficient trans-temporal window for TCD examination. 5. Sufficient trans-orbital window for TCD examination

**Table 2:** The exclusion criteria for diabetic patients and normal volunteers in this study

Exclusion criteria	
1. Penile anatomical defect.	11. History of alcohol or drug abuse.
2. History of priapism.	12. Hypotension (BP < 90/60 mmHg)
3. History of prostatectomy	13. Uncontrolled hypertension (BP > 170/100)
4. History of cerebrovascular disease transient ischaemic attack (TIA), complete stroke or extracranial or intracranial steno-occlusive lesions or altered cerebral hemodynamic.	14. Proliferative retinopathy, retinitis pigmentosa.
5. Major hematological, renal, or Hepatic disorder.	15. Autonomic neuropathy
6. Any major psychiatric disorder	16. History of ketoacidosis in the previous 2 years
7. Treatment with nitrates	17. History of asthma or chronic obstructive pulmonary disease (COPD).
8. Coronary artery disease	18. Recent abdominal surgery.
9. History of myocardial infarction (MI).	19. Eye disease like cataract, glaucoma, intra-ocular lens implantation.
10. Peptic ulcer disease.	20. Diabetic nephropathy.

### Protocol of Transcranial Doppler examination

TCD examination was conducted in a quiet temperature - controlled room at 9:00 am, after an overnight fast. Siemens Versa plus Sonoline equipment (230 PAL versions, Germany) with Color flow and TCD facilities was used for this purpose; a phase array probe of 2-2.5 MHz was used to insonate the MCA. All subjects were allowed to rest quietly for at least 10 minutes to achieve a steady state of heart rate and blood pressure. Before proceeding to the definitive recordings, subjects were trained to perform the procedure of breath hold and hyperventilation correctly. Blood pressure and heart rate were recorded, using mercurial sphygmomanometer ALPK2, and digital infra-red pulse-oximeter respectively. To study the MCA blood flow velocities, the subject was asked to tilt his head to a side and to breath normal quiet breathing, the MCA was first identified (the proximal portion), by color flow, utilizing a trans-temporal window. To obtain MCA spectral wave form, pulse wave (PW) Doppler sample volume was place in the middle of the artery, at an angle of 0° and the means of PSV, DV and RI of 10 consecutive cardiac cycles were recorded. To demonstrate the response of MCA flow to hypercapnea, the subject was asked to hold breath, as long as possible, after mild to moderate deep inspiration to avoid suffocation; the changes of mean flow velocity of MCA to breath hold (BH_mca_) was calculated as the mean of each of the above cited parameters during the last 5 sec. To demonstrate the response of MCA to hypocapnea, the subject was asked to perform moderate hyperventilation for 1.5 min, Doppler spectral parameters of changes in MCA flow and the mean flow velocity of MCA to hyperventilation (HV_mca_) were calculated in the same for mentioned manner.

A 50 mg of sildenafil was then given orally; a period of 50 minutes was allowed to elapse to ensure maximal effect of the drug. Another Doppler waveform baseline control of MCA was taken and all maneuvers to elicit MCA vasoreactivity were repeated in the same fashion cited above.

### Calculation of Doppler Indices

1. Resistive index (RI) [12]

RI was calculated from the following equation

$$\text{RI = }\left[\text{peak systolic velocity }\left(\text{PSV}\right)-\text{end diastolic velocity }\left(\text{EDV}\right)\right]/)\text{PS}$$

2. Mean flow velocity (MFV) [13]: The mean flow velocity baseline control of MCA (MFV_mca_) was calculated as[13]

$$\text{MFV = }\left(\text{PSV + 2DV}\right)/)3$$

3. Breath hold index (BHI): The breath hold index (BHI) is calculated as

$$\text{BHI = }\left[\left(\text{MF}{\text{V}}_{\text{BH}}-\text{MF}{\text{V}}_{\text{baseline}}\right)/)\text{MF}{\text{V}}_{\text{baseline}}\right]\times 100/)\text{seconds of breath hold }\left(4\right)$$

4. Full range of vasodilatation (FVD): The full range of vasodilatation is calculated according equation Markus & Harrison(1992)

$$\text{FVD = }100\times \left[{\text{V}}_{\text{mca}}\left(\text{hypercapnea}\right)-{\text{V}}_{\text{mca}}\left(\text{hyperventilation}\right)\right]/){\text{V}}_{\text{mca}}\left(\text{rest}\right)$$

### Statistical analysis

Data were expressed as mean ± SEM. Paired t-test was used to compare the difference between pre- and post treatment of each group. Independent t-test was used to compare the difference between diabetic and normal groups. Statistical analysis was performed using computer program (SPSS), version 12. In all tests P value < 0.05 was considered to be statistically significant.

## Results

In all 35 participants, there were no statistically significant differences in the baseline demographic and biochemical characteristics regarding age, body mass index(BMI), serum triglyceride, serum VLDL and serum uric acid (p > 0.05). Fasting serum glucose was significantly higher in diabetic group than in normal group (p < 0.05). Serum cholesterol, LDL and atherogenic index were significantly higher in diabetic group than in normal group (p < 0.05) while serum HDL was lower in diabetic patients (table 3).

**Table 3:** Baseline demographic and biochemical characteristics

Character	Diabetic patients	Normal group
**Age (years)**	50 ± 1.35	45 ± 2.17
**Fasting serum glucose mg/dl**	158.4 ± 10.11	87.8 ± 2.92*
**Serum cholesterol mg/dl**	198.7 ± 13.55	173 ± 5.67*
**Serum triglyceride mg/dl**	166 ± 16.8	167.4 ± 11.2
**Serum HDL mg/dl**	34.2 ± 2	48 ± 1.43*
**Serum LDL mg/dl**	130.8 ± 13.1	91.5 ± 5.20*
**Serum VLDL mg/dl**	33.2 ± 3.36	33.48 ± 2.23
**Atherogenic index (AI)**	4.78 ± 0.29	2.64 ± 0.17*
**Serum uric acid mg/dl**	4.51 ± 0.37	4.38 ± 0.39
**BMI**	26.1 ± 0.72	26.02 ± 0.78

*P < 0.05

### Effect of sildenafil on mean flow velocity (MFV) of MCA in normal subjects and diabetic groups

**Figure 1:**
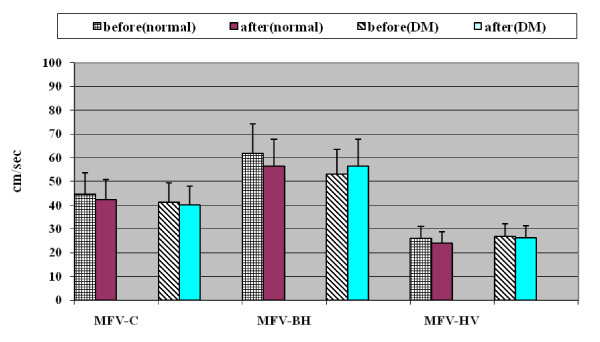
**Effect of sildenafil on MFV of MCA in normal individual and diabetic patients**.

**Figure 2:**
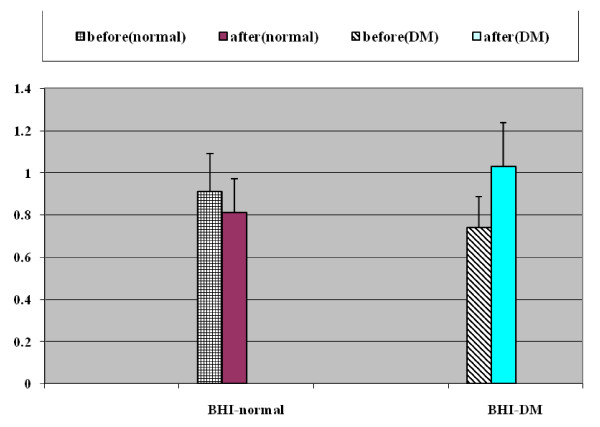
**Effect of sildenafil on BHI in normal persons and diabetic patients**.

**Figure 3:**
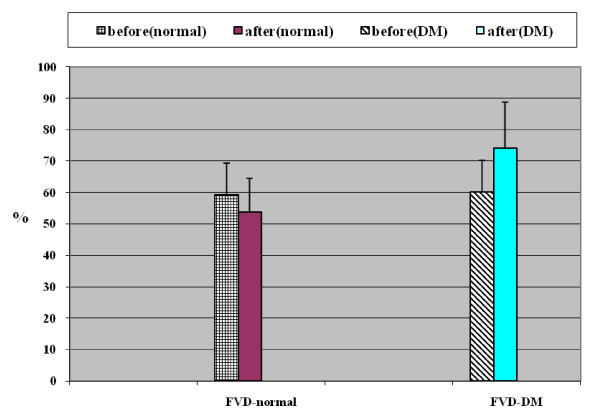
**Effect of sildenafil on FVD of MCA in normal persons and diabetics**.

**Figure 4:**
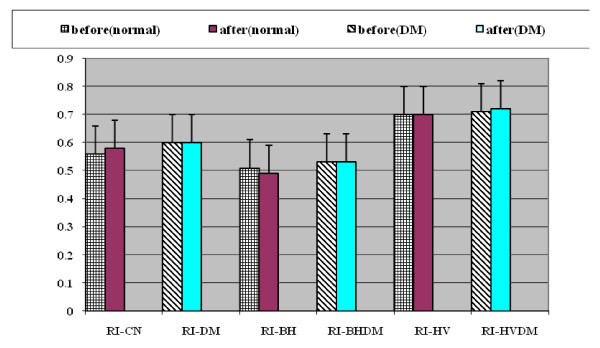
**Effect of sildenafil on resistive index of MCA**. RI: resistive index, C: control, N:normal persons, DM: diabetes mellitus, BH: breath hold, HV: hyperventilation.

**Figure 5:**
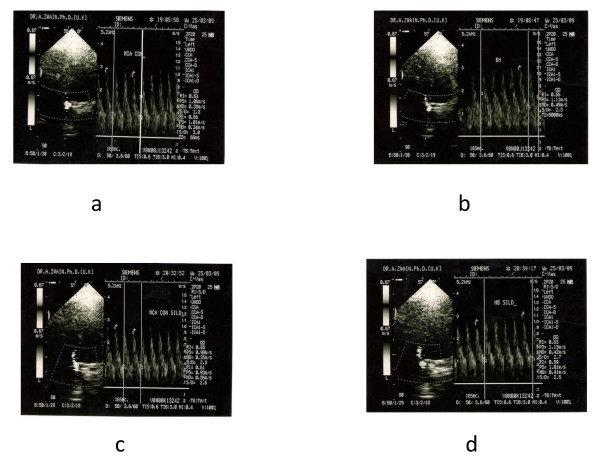
**Doppler spectral wave form during insonating of MCA in diabetic patient before sildenafil (a), (b) and after sildenafil (c), (d) **. There was an apparent difference in MCA flow velocity between before and after giving sildenafil.

Breath holding significantly increased MFV of MCA in both normal subjects and diabetic patients before and after giving sildenafil (p < 0.05). Hyperventilation significantly decreased MFV in both normal subjects and diabetic patients before and after sildenafil (p < 0.05); there was no statistically significant change (increase or decrease) of MFV, after sildenafil during baseline, breath hold/hyperventilation maneuver, in comparison to those before sildenafil, in both normal and diabetic groups(p > 0.05) (Table 4 Figures 1, 2, 3, 4 and 5).

**Table 4:** The effect of sildenafil on MFV (cm/sec) of MCA

Group	MFV before sildenafil			MFV after sildenafil		
	
	Control	Breath hold	Hyperven-Tilation	Control	Breath hold	Hyperven-Tilation
**Normal**	44.7 ± 2.09	61.9 ± 5.02*	26 ± 1.72*	42.5 ± 2.71	55.8 ± 3.68*	24 ± 1.57*

**DM**	41.2 ± 3.35	53 ± 4.64*	26.9 ± 2.36*	40 ± 3.42	56.5 ± 5.02*	26.2 ± 2.39*

*P < 0.05

### Effect of sildenafil on breath hold index (BHI) and full range of vasodilatation (FVD) in normal individuals and diabetics

There was no statistically significant change in BHI and FVD after sildenafil in normal individuals. There was a statistically significant increase in BHI and FVD (p < 0.05) after sildenafil in diabetic patients (see table 5 and Figures 2, 3, 4 and 5).

**Table 5:** Effect of sildenafil on BHI and FVD of MCA

Group	Breath Hold Index (BHI)		Full Range of Vasodilatation (FVD)	
	
	Before sildenafil	After sildenafil	Before sildenafil	After sildenafil
**Normal**	0.91 ± 0.11	0.81 ± 0.09	59.4% ± 6.3%	53.7% ± 4.9%

**DM**	0.74 ± 0.14*	1.03 ± 0.14*	60.2% ± 4.96%*	74% ± 4.8%*

*P < 0.05

### Effect of sildenafil on resistive index (RI) in normal and diabetic individuals

There was statistically significant decrease in resistive index (RI) of MCA in both normal and diabetic groups during breath hold before and after sildenafil (p < 0.05).

During hyperventilation, RI showed statistically significant increase in normal individuals and diabetic patients before and after sildenafil (p < 0.05). Comparison of the effect of sildenafil on RI between normal and diabetic groups revealed no statistically significant change (during baseline, breath hold and hyperventilation (table 6 and Figures 4, 5 and 5).

**Table 6:** Effect of sildenafil on RI of MCA

Group	Resistive Index before sildenafil			Resistive Index after sildenafil		
	
	Control	Breath hold	Hyperventilation	Control	Breath hold	Hyperventilation
**Normal**	0.56 ± 0.02	0.51 ± 0.02*	0.7 ± 0.03*	0.58 ± 0.02	0.49 ± 0.02*	0.7 ± 0.02*

**DM**	0.6 ± 0.02	0.53 ± .02*	0.71 ± 0.02*	0.6 ± .015	0.53 ± .02*	0.72 ± .018*

*P < 0.05

## Discussion

In a randomized, double-blind placebo controlled study of twenty-eight men with erectile dysfunction; Diomedi et al [11] reported that sildenafil had no effect on cerebral mean blood flow velocity. However, using breath holding index and transcranial Doppler to assess the endothelial response to hypercapnea, they demonstrated that a single dose of 50 mg of sildenafil produced an improvement in cerebrovascular reserve. Though this may reduce the concern for the use of sildenafil in patients at risk for cerebrovascular disease, it may increase the concern in migraine, a common adverse effect of sildenafil. On the ground of the above cited conflicting reports, further studies are warranted to verify whether or not sildenafil exert a modulatory effect on cerebral vasoreactivity, bearing in mind a paucity of reports exists on this aspect, regarding patients with type2 DM [11]. The present investigation was aimed to evaluated the acute modulatory effects of sildenafil on cerebral hemodynamic. Our findings showed that 50 mg oral sildenafil did not produce a significant increase of mean flow velocity of MCA at rest in both normal and diabetics. RI did not change significantly after sildenafil; these results are in accordance with previous studies focusing on lack of significant changes in CBF velocity after the administration of 100 mg sildenafil [10,14]. In the present investigation, it was demonstrated that sildenafil produced a significant increase in cerebrovascular reactivity measures (BHI and FVD) in patients with type 2 diabetes mellitus without significant change in other hemodynamic. These results are identical with those reported by Diomedi et al [11], who found a significant improvement in cerebrovascular reactivity after 50 mg of sildenafil in patients with erectile dysfunction. Accordingly, they suggested that sildenafil increased the sensitivity of endothelial cells to hypercapnic stimuli through activation of NO synthase. Kruuse et al [10] found that inhibition of phosphodiesterase-5 enzyme induced relaxation of isolated cerebral arteries, whereas no change in cerebral blood flow was reported after non-PDE selective or PDE-5-selective inhibition [10,15]. On the other hand, other studies showed a favorable acute and prolonged effect of sildenafil on brachial arterial flow-mediated dilatation (an endothelial-dependent dilatation) in patients with impaired endothelial function due to type 2 diabetes and in patients with heart failure [16,17]. This effect may be explained as phosphodiesterase -5 inhibitors could play a role in activating endothelial NO synthase, increasing the sensitivity of endothelial cells to vasodilatory stimuli; single dose of sildenafil can reverse chronic endothelial dysfunction [18]. In normal subjects, sildenafil did not increase BHI neither changed MFV RI nor PI. These results are consistent with previous studies demonstrated that sildenafil did not improve flow-mediated brachial artery dilatation in healthy subjects [19]. This diversity of the effect of sildenafil on diabetic and healthy subjects cerebral reactivity may reflect better improvement or perhaps amelioration of endothelial dysfunction in diabetic patients after sildenafil. A possible explanation of the current observation is furnished in the following argument: Sildenafil may decrease endothelin-1, a potent vasoconstrictor peptide, (ET-1) activity which is greater in diabetics than in healthy subjects; it was reported that blockade of endothelin receptor (ETA), which transudes the biological effects of ET-1, results in vasodilatation in patients with diabetes but not in control group [20]Moreover, Gilbert and co workers [21] found that Endothelin-1 reduces cerebral artery sensitivity to nitric oxide and blockade of (ETA) receptor sensitizes vascular smooth muscle to exogenous and endothelium-derived NO. More relevant data to support our findings was provided by several studies [17] observed that cyclic GMP decreased in those with endothelium dysfunction due to decreased synthesis of cGMP and increased degradation of cGMP by phosphodiesterase or downstream abnormalities in the cGMP signal transduction pathway. Thus, it can be said that sildenafil, as PDE-5 inhibitor, increases the bioavailability of cGMP and consequently improves of endothelial dysfunction. Fisher et al [22]; Lincoln [23] provided evidence that sildenafil stimulates the synthesis of inducible nitric oxide synthase (iNOS) and eNOS mRNA in cardiac myocytes, resulting in increased NO generation, guanylate cyclase activation and enhanced formation of cGMP. However, there appears to be a great controversy on the effect of sildenafil on cerebral vasculature endothelial dysfunction. Dishy et al [21] concluded that sildenafil did not potentiate endogenous NO-mediated vascular responses in forearm conduit or resistance vessels in chronic smokers. In another study, it was concluded that sildenafil did not improve peripheral endothelium dependent vasomotor or fibrinolytic function in patients with coronary heart disease; Phosphodiesterase type 5 inhibitors were unlikely to reverse the generalised vascular dysfunction seen in patients with coronary heart disease [19]. Further study, with larger sample size, is therefore recommended, to support our fore mentioned findings.

## Conclusion

Sildenafil improves cerebral vasoreactivity in type 2 diabetic patients but not in normal individuals. In this account, an oral administration of low dose of PDE-5 inhibitors may be favored in these patients, to improve endothelial function and hence CVR. Sildenafil has no appreciable effect on MFV of MCA baseline mean velocity blood flow in both normal subjects and diabetic patients.

## Competing interests

The authors declare that they have no competing interests.

## Authors' contributions

FG conceived of the study, and participated in its design and coordination. AA participated in Doppler examination, the design of the study and performed the statistical analysis. NR participated in the sequence alignment and drafted the manuscript, design and coordination. AM carried out most of the animal research work and lab measurement. All authors read and approved the final manuscript.
